# Impact of a personal learning plan supported by an induction meeting on academic performance in undergraduate Obstetrics and Gynaecology: a cluster randomised controlled trial

**DOI:** 10.1186/s12909-015-0325-2

**Published:** 2015-03-11

**Authors:** Richard P Deane, Deirdre J Murphy

**Affiliations:** Department of Obstetrics & Gynaecology, Trinity College, University of Dublin, Coombe Women & Infants University Hospital, Dublin 8, Republic of Ireland

**Keywords:** Learning plan, Induction meeting, Undergraduate, Obstetrics & Gynaecology, Academic performance

## Abstract

**Background:**

A personal learning plan (PLP) is an approach to assist medical students maximise their learning experience within clinical rotations. The aim of this study was to investigate whether medical students who created a PLP supported by an induction meeting had an improved academic performance within an undergraduate clinical rotation.

**Methods:**

A cluster randomised controlled study was conducted over a full academic year (2012/13). The intervention was the creation of a PLP by medical students supported by an individual ‘one-to-one’ induction meeting between each student and a faculty member. Randomisation was by unit of rotation in which students completed the program. There were 2 clusters in the intervention group (n = 71 students) and 2 clusters in the control group (n = 72 students). Primary outcome was the overall examination score. Secondary outcomes were student attendance and student evaluation.

**Results:**

There was no difference in overall examination score between the intervention group and control group (mean score 56.3 ± 4.8% versus 56.7 ± 5.6%, p = 0.64). The majority of students in the intervention group (n = 51/71, 85%) reported that the PLP and induction meeting enhanced their learning experience. Attendance at the induction meeting was identified as a key element.

**Conclusions:**

The creation of a PLP supported by an induction meeting was rated highly by students as an approach to enhance their learning experience but did not result in an improved academic performance. Further research is required to establish the role of an interim or exit meeting.

## Background

The use of a personal learning plan (PLP) in postgraduate medical education is well established as an integral part of developing and maintaining professional competence. However, the role of PLPs in undergraduate medical education is less clear. The Association for Medical Education in Europe (AMEE) published a comprehensive review of PLPs in medical education and highlighted the potential application within undergraduate medical education [1]. There are a small number of studies that have found a benefit to goal setting by medical students within clinical specialties [2-5]. The use of PLPs within the clinical learning environment is particularly attractive as medical students often struggle with adapting to learn within this less familiar and structured environment [6]. PLPs may offer an approach to assist students in developing the ‘adult’ learning approaches required within the clinical setting [1].

This study is based on the hypothesis that the creation of a PLP by medical students supported by an induction education meeting, similar to the approach taken in postgraduate training, improves their academic performance. The primary aim of this study was to investigate whether medical students who created a PLP supported by an individual ‘one-to-one’ induction education meeting had an improved academic performance within an undergraduate clinical rotation in Obstetrics and Gynaecology (O&G). There were a number of unique elements to the study in comparison with the existing published studies on PLPs [2-5]. Firstly, the study used a ‘one-to-one’ meeting with a faculty member to support the creation of the PLP. Secondly, in addition to setting specific learning goals, the PLP addressed the learning approaches by medical students to the clinical rotation as a whole.

## Methods

### Study setting

The study was conducted within the Department of Obstetrics and Gynaecology, Trinity College, University of Dublin, Ireland. The undergraduate programme in O&G is completed over 8 weeks in the penultimate year of the 5-year degree course in medicine. There are 4 rotations during the academic year. The programme consists of a combination of clinical and tutorial-based learning activities. The assessment modalities used to determine the overall examination score are an end-of-rotation 11 station objective structured clinical examination (OSCE) (20%) and an end-of-year examination consisting of 50 single best answer questions (SBAs) (20%), 6 modified essay questions (MEQs) (20%) and a long case clinical examination (40%). Students require an overall examination score of 50% to pass and 60% to be awarded a distinction.

### Study design

The study was designed as a 4-group cluster randomised controlled trial (RCT) during the 2012/13 academic year. Cluster randomisation was adopted as individual randomisation within each rotation may have led to contamination between students. The class of 145 students was divided by administrative staff within the medical school into 4 groups ensuring that the rotations were of similar demographic distribution. Each of the 4 rotations during the year was defined as a separate cluster. Each cluster was randomised to either the intervention group (received the PLP and induction meeting in addition to the routine introductory presentation) or the control group (received the routine introductory presentation alone). Simple randomisation was used in a 1:1 allocation ratio using computer random number generation. Rotations 2 (Nov/Jan) and 3 (Feb/Mar) were allocated to the intervention group and rotations 1 (Sep/Oct) and 4 (Apr/May) were allocated to the control group. Only the academic staff member conducting all of the induction meetings and the student participating in the induction meeting were aware of the student’s participation. Institutional Research Ethics Board approval for the study was obtained (TCD Research Ethics Committee, approval October 2012).

### Participants

The study was conducted among the entire class completing their O&G rotation during the 2012/13 academic year. There was no restriction of participants with all rotations during the year and all students within each rotation eligible for inclusion. Although the sample size was dictated by the class size, a power calculation indicated that this sample size was sufficient to detect a difference of 5 percent in overall examination score between the groups (assuming power of 0.8 and significance level of 0.05). An information leaflet on the study was sent to students allocated to the intervention group by email 1 week prior to starting their rotation. Students were asked to complete and submit a consent form to indicate whether they wished to participate in the study on the first day of the rotation. Following receipt of the consent forms, the supervisor sent each student who consented a time for his/her meeting.

### Intervention

The intervention was the creation of a PLP by the medical student supported through an individual ‘one-to-one’ induction education meeting between the student and an academic staff member during the first 2 weeks of the clinical rotation. The control group received a group presentation lasting approximately 20 minutes on how to optimise their learning experience during the rotation. The teaching programme within each rotation was otherwise the same.

The PLP was an 8-page handbook divided into 6 sections identified as imperative for the construction of a PLP by the AMEE Guide [1]. The PLP was constructed using a variety of open-ended and closed questions. Part 1 (Importance of the O&G Rotation) required students to reflect on the importance of the O&G rotation. Part 2 (Relevance of the O&G Rotation for Future Careers) required students to formulate a specific and practical learning outcome to be achieved that would be beneficial in his/her future career. Part 3 (Academic Targets) required students to define a specific academic target by considering typical self-reported academic performance. Part 4 (Learning Resources) required students to identify their main learning resource by rating a series of commonly used resources during the rotation and selecting a resource for the rotation. Part 5 (Study Schedule) required students to create a study plan documenting the topics to be covered each week. Part 6 (Learning Activities) required students to identify strategies to maximise their learning experiences in the clinical learning environment. The booklets were piloted in August 2012 among students from the previous academic year.

The purpose of the induction meeting was to support the creation of the PLP. The PLP created by the student was reviewed at the meeting (as each student was asked to complete the PLP in advance of the induction meeting). The supervisor explored and clarified each section of the PLP with student. In addition, the supervisor suggested a range of possible learning strategies for the student to consider. The supervisor adopted a ‘questioning’ style (rather than a ‘didactic’ style) in order to allow the student to create his/her own PLP consistent with the principles of good supervision technique [7]. The benefits and drawbacks of the various approaches suggested by students were discussed and students were free to add to or amend the learning plan during the induction meeting. The same staff member (RPD), an experienced medical educator familiar with the course programme, conducted each meeting to ensure consistency. At the end of the induction meeting each student had a completed PLP for the clinical rotation.

### Outcomes

The primary outcome was the overall examination score obtained by the student. The secondary outcomes were student attendance (defined as a percentage of the total possible attendance at all scheduled clinical and classroom-based activities) and student evaluation of the supported PLP.

### Data collection

Data related to student demographics and academic performance were obtained from the departmental records linked by code to ensure confidentiality. The data relating to student attendance were based on staff signatures in the student logbook. The student evaluation survey was completed using an online survey tool (SurveyMonkey®) approximately 1 month following the rotation.

### Data analysis

Student demographics were compared using chi-squared tests and Fisher’s exact tests. Examination scores and attendance rates between the intervention group and the control group were compared using an unpaired *t*-test. Students who responded to the survey were compared with non-responders using chi-squared tests. Content analysis was used to identify themes from the free text questions on the student survey. SPSS version 19 was used for statistical analysis. A significance level of 0.05 was used.

## Results

### Participation

**Figure 1:**
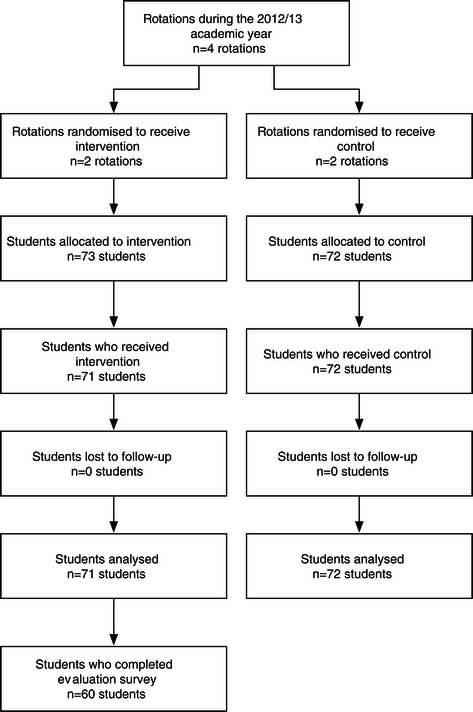
**Flow diagram.**

A total of 145 students completed the undergraduate programme in O&G during the 2012/13 academic year. There were 72 students allocated to the control group and 73 students allocated to the intervention group (Figure 1). There were 2 students allocated to the intervention group who declined to participate leaving 71 students. A demographic profile of the students is provided in Table 1. A total of 71 PLPs were completed and 71 meetings were conducted. The total face-to-face meeting time was 37 hours with a mean duration for each meeting of 31 minutes ± 9 minutes (ranging from 15 to 60 minutes).

**Table 1:** **Student demographics**

	Intervention group n = 71	Control group n = 72	p value
**Gender**			
Male	39 (55%)	38 (53%)	0.80
Female	32 (45%)	34 (47%)	
**Age**			
20–24 years	57 (80%)	50 (70%)	0.10
25–29 years	9 (13%)	19 (27%)	
≥30 years	5 (7%)	3 (3%)	
**Country of origin**			
European Union	46 (64%)	55 (78%)	0.07
North America	16 (22%)	6 (9%)	
Asia/Africa	10 (14%)	10 (13%)	
**Previous end-of-year failure**			
Yes	23 (32%)	17 (24%)	0.24
No	48 (68%)	55 (76%)	

### Academic performance and attendance

There was no significant difference in mean overall examination score between the groups; 56.3 ± 4.8% in the intervention group and 56.7 ± 5.6% in the control group (p = 0.64) (Table 2). The mean total attendance rate was 86.7 ± 9.0% in the intervention group and 88.4 ± 8.7% in the control group (Table 2). There was no significant difference in overall attendance between the groups (p = 0.25) although attendance at clinical activities was more likely in the intervention group (p = 0.03) and attendance at classroom-based activities more likely in the control group (p = 0.01).

**Table 2:** **RCT findings for academic performance and attendance**

	Intervention group n = 71	Control group n = 72	p value
	Mean % (SD), range	Mean % (SD), range	
Overall examination score	56.3% (4.8), 45–70	56.7% (5.6), 44–73	0.64
OSCE score	56.6% (5.9), 45–70	56.2% (5.8), 45–70	0.65
MCQ score	59.2% (8.1), 42–75	59.6% (8.2), 42–75	0.79
SAQ score	52.3% (5.2), 30–60	52.4% (4.6), 40–65	0.90
Clinical examination score	55.9% (5.4), 43–68	56.7% (7.2), 37–72	0.43
	Number of students (%)	Number of students (%)	
Fail grade	5 (7%)	4 (5%)	0.90
Pass grade	48 (68%)	48 (67%)	
Distinction grade	18 (25%)	20 (28%)	
	Mean % (SD), range	Mean % (SD), range	
Total attendance	86.7% (9.0), 45–100	88.4% (8.7), 51–100	0.25
Clinical attendance	91.2% (9.2), 42–100	87.5% (10.9), 38–100	0.03
Tutorial-based attendance	84.0% (11.3), 47–100	88.8% (10.4), 49–100	0.008
Attendance categories	Number of students (%)	Number of students (%)	
0 to 69% attendance	1 (2%)	2 (3%)	0.27
70 to 79% attendance	10 (14%)	4 (6%)	
80 to 89% attendance	26 (37%)	34 (47%)	
90 to 100% attendance	33 (47%)	32 (44%)	

### Student survey

The response rate for the student evaluation survey was 85% (n = 60/71). There was no significant difference between responders and non-responders in terms of demographic profile, academic performance or attendance. Tables 3 and 4 provide a summary of the responses to the quantitative questions. The majority of students recommended that the best way for staff to support students in developing their learning plans was a ‘one-to-one’ meeting with a supervisor and a follow-up meeting in the rotation (n = 39, 65%). A number of students recommended an interactive discussion amongst a small group of students (n = 10, 16%) or single ‘one-to-one’ meeting (n = 9, 15%). Only a single student recommended a large group presentation (n = 1, 2%) or no support activity (n = 1, 2%). Student opinion regarding the most appropriate time to introduce these meeting was divided with 32 students (54%) advising introduction during the clinical years (year 4-5), 23 students (38%) preferring introduction during the pre-clinical years (year 1-3) and 5 students (8%) had no opinion.

**Table 3:** **Student responses to survey**

	Yes *n* (%)	No *n* (%)	Don’t know *n* (%)
Was this type a meeting a new experience for you a medical student?	57 (95)	2 (3)	1 (2)
Did the information you received from the learning plan and the meeting enhance your learning experience during the rotation?	51 (85)	4 (7)	5 (8)
Did you attend more during the rotation as a result of the information you received from the learning plan and the meeting?	23 (38)	26 (43)	11 (19)
Did you follow your learning plan during the rotation?	42 (70)	18 (30)	0 (0)
Would you have found a follow-up meeting later in the rotation helpful?	44 (73)	5 (9)	11 (18)
Would it be worthwhile offering a similar meeting to students completing the O&G rotation in the future?	58 (96)	1 (2)	1 (2)
Would it be worthwhile introducing a similar meeting for students completing rotations in other clinical specialties?	59 (98)	0 (0)	1 (2)
Is it reasonable to expect students completing their O&G rotation to prepare and submit a learning plan?	43 (71)	10 (17)	7 (12)

**Table 4:** **Student ratings for the PLP/meeting components**

	Very unhelpful (1) *n* (%)	Unhelpful (2) *n* (%)	Neither (3) *n* (%)	Helpful (4) *n* (%)	Very helpful (5) *n* (%)	Mean score	Rank order
Part 1: importance of the O&G rotation	0 (0)	0 (0)	14 (23)	43 (72)	3 (5)	3.8	5=
Part 2: relevance of the rotation for future careers	0 (0)	0 (0)	15 (25)	36 (60)	9 (15)	3.9	4
Part 3: academic targets	0 (0)	0 (0)	4 (7)	38 (63)	18 (30)	4.2	2
Part 4: learning resources	0 (0)	1 (2)	8 (13)	31 (52)	20 (33)	4.2	1
Part 5: learning activities	0 (0)	4 (7)	11 (18)	37 (62)	8 (13)	3.8	5=
Part 6: study schedule	0 (0)	1 (2)	9 (15)	33 (55)	17 (28)	4.1	3

The main themes from the qualitative analysis of the free text questions are highlighted in Table 5. Students identified the aspects of the intervention that worked well: use of an induction meeting to create the PLP (Student 11); ‘one-to-one’ nature of the induction meeting (Student 60); provision of the meeting early in the rotation (Student 50); identification of expectations and goals (Student 40); positive nature of the meeting (Student 6). Students identified the aspects of the intervention that could be improved: provision of an interim or exit meeting (Student 21); incorporation of more advice from lecturers and past students (Student 66); use of a small group format with other students (Student 51). Students identified the main difficulties encountered in adhering to the PLP including lack of familiarity with the course material (Student 62) and mismatching of classroom-based tutorials and clinical activities (Student 15).

**Table 5:** **Qualitative analysis – selected student quotes**

Aspects of the PLP and induction meeting that worked well	Aspects of the PLP and induction meeting that could be improved
“Development of the learning plan. Don’t think I would construct a proper learning plan without the induction meeting”. Student 11: 95% attendance, 53% score.	“I did not have any follow up meeting after the rotation ended so if that'd happen it would be very useful”. Student 21: 98% attendance, 55% score.
“One on one means you can talk openly”. Student 60: 96% attendance, 58% score.	“Perhaps more tips from previous students who did well”. Student 66: 90% attendance, 51% score
“Often you only realise what is expected of you halfway through a rotation, so this meeting gave 1-2 weeks headstart. Would be useful to have in all clinical rotations”. Student 50: 95% attendance, 59% score.	“It may have worked better in a small group setting, just because that would encourage the setting up of study groups amongst students, and also students could learn from the studying methods of their peers”. Student 51: 80% attendance, 57% score.
“It gives a focus to what needs to be done and makes the goals seem achievable”. Student 40: 83% attendance, 56% score.	“My learning plan was too ambitious, hard to judge before knowing much about the course”. Student 62: 74% attendance, 52% score.
“It was so positive, an individualised pep talk. The information on clinical learning was extremely useful outside of obs/gyne too”. Student 41: 94% attendance, 58% score.	“It was difficult to stick to the learning plan, when the lecture topics and what week of the clinical O + G rotation you were on did not correspond!” Student 15: 95% attendance, 61% score.

## Discussion

The creation of a PLP supported by an individual ‘one-to-one’ induction education meeting was rated highly by students as an approach to enhance their learning experience but did not improve their academic performance or attendance. Students reported that an interim or exit meeting might have helped in the application of the learning plan. There are a number of key questions that arise from the literature for medical educators considering the introduction of PLPs for undergraduate medical students: What should PLPs target? Who should PLPs target? When should PLPs be introduced? What faculty supports are required for PLPs? Are PLPs beneficial? The discussion will consider the current evidence and the findings of this study for each of these key questions.

What should PLPs target? The need for PLPs among postgraduate trainees and specialists is intuitive as they usually self-define learning goals depending on their learning needs and opportunities. In contrast, the need for PLPs among medical students following a structured programme of learning activities to achieve pre-defined learning goals in a specific time frame is less clear. Previous studies have shown that medical students can create specific learning goals within clinical rotations and that PLPs are helpful in assisting students achieve these learning goals [2-5]. However, this study has showed that supported PLPs can be used successfully to plan each student’s overall approach to learning within clinical rotations and not only to achieve specific learning goals. The positive student rating of each of the PLP components, particularly the learning resources and study schedule, highlights this finding.

Who should PLPs target? PLPs can target all students or specific student groups e.g. students in difficulty. The use of remedial teaching programmes for medical students in difficulty is well established [8,9]. These programmes often involve the identification of specific deficits and the planning of strategies to address these deficits. Therefore, individual educational direction within clinical rotations is often provided for a small number of students at the extremes of academic ability but not for the majority of students. The approach in this study was to offer the PLP and induction meeting to all students. The student survey supported this approach with 71% (n = 43) responding that it is reasonable to expect students to prepare and submit a learning plan. In addition, student participation was voluntary and the high participation rate (97%, n = 71) suggests that students of all backgrounds are willing and wish to engage with PLPs.

When should PLPs be introduced? The optimal timing for the introduction of PLPs requires consideration: where within the medical course as a whole and within specific clinical rotations? This study suggests that students are divided on whether the supported PLPs should be introduced during pre-clinical years (n = 23, 38%) or clinical years (n = 32, 54%). However, the small majority in favour of clinical years likely reflects the well-documented challenges many students encounter adapting to the clinical learning environment [10]. Within the clinical rotation itself, students recommended that the PLP and induction meeting should take place early in the rotation. However, students also acknowledged that some of the difficulties experienced in following the PLPs were due creating the PLP early in the rotation and the consequent lack of familiarity with the course material. Therefore, the introduction of PLPs during clinical years and early within clinical rotations appears optimal. However, students need to be provided with a clear identification of the knowledge and skills that must be acquired in order to create viable and effective PLPs.

What faculty supports are required for PLPs? The AMEE review suggests that PLPs should be created with supervisory support [1]. In contrast to previous PLP studies with variable amounts of supervisory support, a unique feature of this study was the explicit use of an induction meeting to support the creation of the PLP [2-5]. The student survey identified that the ‘one-to-one’ meeting was a critical and welcome element. However, many students also identified that the use of an interim or exit meeting may have enhanced the intervention. Although this is an attractive prospect, educators need to consider the significant time investment by faculty members to provide this level of individualised educational support. Therefore, the provision of ‘one-to-one’ supervisory support is an important element in the use of PLPs but this support may need to be ongoing.

Are PLPs beneficial? In contrast to previous PLP studies that only evaluated benefit using student surveys i.e. evaluation of reaction, this study also evaluated benefit using academic performance (as the primary outcome) i.e. evaluation of learning [2-5]. There was no difference in the academic performance between the groups. However, like previous PLP studies, the student satisfaction with the intervention was high: students reported that the PLP and the induction meeting enhanced their learning experience (n = 51, 85%), they used their PLPs (n = 42, 70%) and recommended that a similar intervention should be provided in O&G and other clinical specialties (n = 59, 98%). The question arises as to why the evidence of benefit from the student evaluation did not translate into an improved academic performance. There were 2 main reasons. Firstly, the use of academic performance as a primary outcome may have placed too great an emphasis on an objective outcome and may not have fully captured the benefits accrued. Secondly, the impact may have been limited by the lack of an interim or exit meeting to consolidate the PLP. Therefore, ongoing support may be an approach to produce an enhanced academic performance.

### Strengths

The inclusion of the entire class within the study and the high participation rate within the intervention group ensured that the findings are broadly generalisable to other institutions with a similar student demographic and programme design. The use of cluster sampling rather than individual sampling minimised contamination between the intervention and control groups. The blinding of academic staff members minimised observation bias.

### Limitations

The use of an RCT with an objective outcome provided a robust assessment of the proposed intervention but may not have reflected the full range of its potential benefit. Given that the study was performed in a single institution within a single discipline, the findings would need to be replicated in other disciplines and institutions. A cost-effectiveness analysis would inform the debate on whether faculty members’ time should be diverted to this approach.

### Implications for academic practice

Inherently medical educators want their students to consider and plan their learning approaches. Supported PLPs enable medical students and their educators to achieve this. This study shows that supported PLPs are of some benefit but that further research is required on the optimal strategies that student should adopt and the amount of support that faculty should provide.

## Conclusions

The creation of a PLP supported by an individual ‘one-to-one’ induction education meeting was rated highly by students as an approach to enhance their learning experience but did not result in improved academic performance. Further research is required on the optimal approach to incorporating PLPs into undergraduate medical programmes and the amount of support that faculty should provide, particularly in terms of an interim or exit education meeting.
